# Simulating evolutionary responses of an introgressed insect resistance trait for ecological effect assessment of transgene flow: a model for supporting informed decision-making in environmental risk assessment

**DOI:** 10.1002/ece3.463

**Published:** 2013-01-17

**Authors:** Matthias S Meier, Miluse Trtikova, Matthias Suter, Peter J Edwards, Angelika Hilbeck

**Affiliations:** 1 ETH Zurich – Institute of Integrative Biology, Universitätsstrasse 16Zurich, 8092, Switzerland; 2 FiBL – Research Institute of Organic Agriculture, Ackerstrasse 21Postfach 219, Frick, 5070, Switzerland

**Keywords:** Apparent competition, crop–wild gene flow, *Meligethes* beetles, oilseed rape, *Raphanus raphanistrum*, transgenic plants

## Abstract

Predicting outcomes of transgene flow from arable crops requires a system perspective that considers ecological and evolutionary processes within a landscape context. In Europe, the arable weed *Raphanus raphanistrum* is a potential hybridization partner of oilseed rape, and the two species are ecologically linked through the common herbivores *Meligethes* spp. Observations in Switzerland show that high densities of *Meligethes* beetles maintained by oilseed rape crops can lead to considerable damage on *R. raphanistrum*. We asked how increased insect resistance in *R. raphanistrum* – as might be acquired through introgression from transgenic oilseed rape – would affect seed production under natural herbivore pressure. In simulation experiments, plants protected against *Meligethes* beetles produced about twice as many seeds as unprotected plants. All stages in the development of reproductive structures from buds to pods were negatively affected by the herbivore, with the transition from buds to flowers being the most vulnerable. We conclude that resistance to *Meligethes* beetles could confer a considerable selective advantage upon *R. raphanistrum* in regions where oilseed rape is widely grown.

## Introduction

Two issues – gene flow and selective advantage – are central to assessing the consequences of gene escape from transgenic crops to either other crops or wild plants (Manasse 1992). Most studies related to biosafety, however, have concentrated on gene flow, usually with the aim of determining rates of hybridization between crop plants and wild relatives or detecting introgression. In this respect, oilseed rape has been the most intensively studied crop (Jorgensen and Anderson 1994; Lefol et al. 1996a,b; Mikkelsen et al. 1996; Darmency et al. 1998; Warwick et al. 2003), although other species, including sugar beet (Bartsch et al. 1999), rice (Song et al. 2003), wheat (Guadagnuolo et al. 2001; Morrison et al. 2002), and maize (Quist and Chapela 2001) have also been investigated.

These hybridization studies are important because they tell us whether gene flow between related plant species is possible. However, for environmental risk assessment, it is also important to know whether a transgene is likely to confer a selective advantage (or disadvantage) upon a recipient organism, and whether it could have broader ecological consequences for the community (Ellstrand 2003; Hails and Morley 2005). One possible scenario is that transgenes replace wild genes (genetic assimilation), leading to a loss of genetic diversity in wild populations (Levin et al. 1996; Wolf et al. 2001). Indeed, if the hybrids between a transgenic crop and a wild relative are less fertile, transgene flow could cause the populations of the wild relatives to decline (demographic swamping) (Wolf et al. 2001). Alternatively, the hybrids could be more fertile, so that they increase in abundance (Campbell et al. 2006), with potentially harmful consequences for biological diversity or ecosystem integrity (Hooper et al. 2005). It is also possible that transgene flow has no consequences, because the transferred gene is either neutral or detrimental; in the former case, the gene may persist at about its initial frequency, whereas in the latter, it goes extinct (Ellstrand 2003). Assessing which outcome is most likely, however, is difficult, partly because a plant's fitness is determined by interactions with many different organisms, which might be differently affected by the transgene. Furthermore, selection processes usually require a relatively long time to become obvious, making them difficult to assess experimentally. Novel testing procedures are therefore needed to evaluate potential effects of transgene flow.

The aim of this study was to investigate how the fitness of a wild relative would be affected if it acquired increased resistance against an insect herbivore through introgression from a transgenic crop. It follows a system-oriented, environmental risk assessment approach as proposed by Hilbeck et al. (2011), with the GM plant at the center and integrating the organisms that occur in the receiving environment. Most plant species support a variety of herbivores, some of which can strongly reduce plant fitness (Combes 1996), and thus influence the size and distribution of populations (Hilbeck 2001). If a wild relative of a crop plant were to acquire a powerful insect resistance transgene like Bt (from *Bacillus thuringiensis*), we might expect the receiving population to benefit. However, despite the vast areas now planted with transgenic crops (160 million hectares globally in 2011, of which approximately 46% contained Bt transgenes (James 2011)), only two published studies appear to have assessed the consequences for a wild plant species and its associated fauna of acquiring a transgenic insect resistance trait (Snow et al. 2003; Yang et al. 2011). In the first study, a Bt transgene in backcrossed wild sunflower populations led up to a 55% increase in the seeds produced per plant compared with the non-transgenic controls. This effect was primarily due to the additional protection against herbivory conferred by the insecticidal Bt transgene (Snow et al. 2003). In the second study, F2 and F3 crop–weed hybrids of weedy and genetically engineered rice carrying two transgene constructs, a cowpea trypsin inhibitor and a Bt transgene, exhibited up to 79% less insect damage and 47% greater fecundity than non-transgenic controls and a 44% increase in fecundity compared with the weedy parents when target insects were abundant (Yang et al. 2011). Under low insect pressure and direct competition with non-transgenic controls, transgenic F3 hybrids showed only a small fitness cost due to the transgenes.

Using oilseed rape and its wild relative *Raphanus raphanistrum* (Brassicaceae) as a model system, we investigated a scenario in which a transgene conferring resistance to coleopteran herbivores was transferred to a *R. raphanistrum* population. Various species of *Meligethes* (Coleoptera: Nitidulidae), especially *M. aeneus* (Hoffman 1999; König and Heitefuss 2000), are important pests of oilseed rape in Europe, and *M. aeneus* is also becoming a problem on oilseed rape in Canada (Mason et al. 2003). Another host of *Meligethes* spp. is *R. raphanistrum* (Tommes et al. 1998), a wild relative of oilseed rape and a potential mating partner (Darmency et al. 1998). The ecological relationship that exists between *Meligethes* spp., oilseed rape and *R. raphanistrum* is an example of apparent competition, in which a focal prey species (*R. raphanistrum*) is linked to an alternative prey species (the crop) via generalist enemies (*Meligethes* spp. and other shared herbivores) (Holt 1984; Holt and Lawton 1994). Although the two prey species do not compete directly for resources, the focal species may suffer because of the large enemy population that develops upon the alternative prey (Bonsall and Hassell 1997). In our study system, oilseed rape crops provide abundant food and breeding sites for *Meligethes* beetles, which may reach very large densities. For example, Hokkanen (2000) found the reproductive success of *M. aeneus* to be twice as high in areas where rapeseed was cultivated as where the only host plants were wild relatives. Thus, cultivated oilseed rape helps to sustain *Meligethes* populations at densities far higher than would be supported by *R. raphanistrum* plants and other wild Brassicaceae alone (Holt and Lawton 1993). *R. raphanistrum* may therefore experience an indirect negative effect by suffering from unusually high herbivore densities, which could lead to the local exclusion of this species (Holt and Lawton 1994).

Because we could not perform experiments using transgenic plants, we simulated the potential protection gained from the transgene by applying insecticide, as has been recommended by Wilkinson et al. (2003) in cases where transgene introgressed recipients are unavailable. In our study area in Switzerland, *Meligethes* beetles can sometimes be found in large numbers on *R. raphanistrum* plants and on related plant species, especially on plants growing beside oilseed rape fields (unpublished data). In a first experiment, we compared seed production of *R. raphanistrum* plants with and without protection from herbivory growing at different distances from commercial oilseed rape fields. In a second experiment, we examined in more detail how resistance to *Meligethes* spp. affected the production of flowers, pods, and seeds of *R. raphanistrum* plants.

## Materials and Methods

### Field experiment I

#### Plant cultivation

*Raphanus raphanistrum* seeds were obtained from UFA seeds, Fenaco Winterthur, Switzerland. Approximately, 1000 seeds were soaked overnight in a solution of deionized water and gibberellic acid (150 mg/L) to break possible dormancy (Cheam 1986). They were then placed in Petri dishes on filter paper soaked with a solution of deionized water and gibberellic acid (200 mg/L), and allowed to germinate in a climate chamber. The climate chamber regime cycled between 22°C/52% relative humidity during daytime and 20°C/50% relative humidity during nighttime, with a day/night cycle of 16-h light and 8-h dark.

After 1 week, seedlings were transplanted into 12-cm pots filled with standard potting soil and grown in the greenhouse for 12 weeks. Plants were watered regularly, but no fertilizer was supplied. Six hundred plants were chosen at random from this material and planted in the field on April 28th and 29th 2008.

#### Experimental set-up

The experiment was set out beside five commercial winter oilseed rape fields (field size of 1–2 ha) in three districts of northern Switzerland: three fields in Rafz (Canton of Zurich), and one field each in Hönggerberg, (Canton of Zurich) and Tegerfelden (Canton of Argovia). Two of the five fields were part of an agri-environment scheme and were not treated with insecticides; the remaining three fields were sprayed with insecticides, which were applied before the experimental plants were set out.

**Figure 1:**
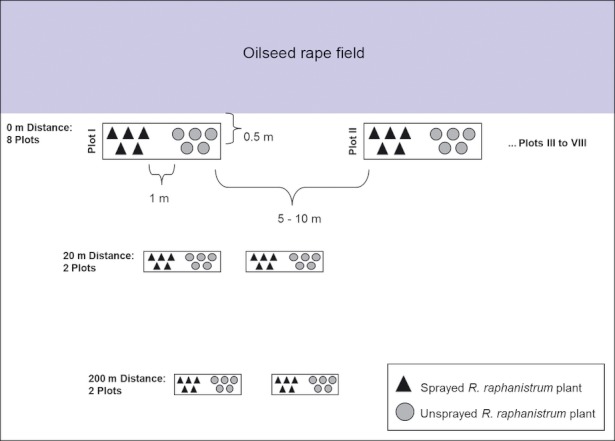
Plot arrangement around oilseed rape fields applied in field experiment I.

At each site, *R. raphanistrum* plants were randomly assigned to one of 12 plots, each with 10 plants (Fig. 1). Eight plots were placed about 0.5 m away from the crop along the edge of the field, and two plots each were placed at distances of approximately 20 and 200 m from the field. The different distances were intended to provide information about the mobility of pollen beetles, with maximum distance of 200 m being determined by the distribution of oilseed rape fields in the landscape. At any one distance, the plots were spaced 5–10 m apart. Five plants in each plot were sprayed weekly with insecticide (0.005% cypermethrin, Sintagro AG, Switzerland) using a hand sprayer, while the remaining five plants, which were 1 m away, served as controls (Fig. 1).

We monitored the numbers of pollen beetles *Meligethes* spp. at weekly intervals on both oilseed rape and *R. raphanistrum* plants. For oilseed rape, beetles were counted on 10 randomly chosen plants along the field edge, using different plants each week. For *R.* *raphanistrum,* the monitoring was performed on three unsprayed and three sprayed plants in two plots at each distance from the field (i.e., a total of 12 plants each at 0 m, 20 m, 200 m). Counting was always carried out prior to spraying and the same plants were used each time. To assess *R. raphanistrum* phenology, we recorded the presence of flower buds, flowers, and pods during the weekly checks. Pods that ripened during the experiment (mature pods) were collected and stored in paper bags.

Surviving *R. raphanistrum* plants (528 in total, Table 1) were harvested from 19–27 June 2008, and the total numbers of mature pods per plant, including any pods that had ripened earlier, were recorded. To estimate the average number of seeds per pod, we counted the seeds in a random subsample of mature pods from the 528 plants (evenly distributed over sites, distances, and treatment) from 147 sprayed and 147 unsprayed plants.

**Table 1:** Total number of *Raphanus raphanistrum* plants analyzed per treatment and distance from oilseed rape fields in experiment I

Distance	U	S	Total
D0	159	173	332
D20	50	49	99
D200	48	49	97
Total	257	271	528

D0 = 0 m, D20 = 20 m, and D200 = 200 m distance from oilseed rape field.

U = unsprayed, S = sprayed.

#### Data analysis

The effect of spraying on the number of mature pods was analyzed with linear mixed regression (Pinheiro and Bates 2000). In this analysis, the response variable was the mean number of mature pods for the five sprayed/unsprayed plants per plot, and the fixed explanatory factors were site, distance from the oilseed rape field, and spraying; plot (Fig. 1) was treated as a random grouping factor. Because distance could not be assigned randomly, a random parameter allowing for the spatial correlation among the distances from field was included in the model. Pod number was log-transformed to meet the assumptions of the model, that is, normal distribution and homogeneity of residual variance. Differences of the response variable among factor levels (site, distance, spraying) were analyzed based on the models' contrasts. All analyses were carried out using the statistics software R (R Development Core Team 2012).

### Field Experiment II

The experiment was conducted in three commercial winter oilseed rape fields (field size = 1–2 ha) in the Rafz district (Canton Zurich, Switzerland). Seeds of *R. raphanistrum* collected from a single mother plant in 2001 were sown in a greenhouse with controlled light and temperature in March 2002. When the plants were at the four- to five-leaf stage, they were planted in pairs 1 m apart in the fields. For each field, four pairs were located at the margin (0.5 m from the crop) and one pair in the center, giving a total of 15 pairs for the three fields. One plant from each pair was treated with insecticide containing 0.5% Chlorpyrifos-ethyl and 0.005% Cypermethrin (Aerofleur® – Maag, Syngenta, Switzerland), which was reapplied each week until the end of the experiment.

#### Parameters measured

Each week from mid-April until mid-July 2002, we recorded the numbers of flower buds, buds that had dropped (determined by the remaining peduncles), open flowers, dropped flowers, pods, and dropped pods (see electronic appendix Table S1). The category “pods” included both mature pods and the styles that persisted after the flowers had withered (immature pods) and subsequently matured into pods. To avoid confusing dropped flowers from dropped pods, flower peduncles were marked with a permanent black marker on the first occasion, and pod peduncles with a red marker. As peduncles of buds could be easily distinguished from peduncles of flowers and pods by their smaller size, no additional marking was necessary. After counting, the peduncles of dropped buds, flowers, and pods were removed to avoid recording them a second time. Mature pods were harvested before they dropped and their seeds were counted. Total numbers of buds, flowers, and pods were calculated based on the recorded parameters (see electronic appendix Table S1) using the formulae listed in Table S2 (electronic appendix). The total number of seeds per plant was determined by cracking all pods of the individual plants and counting the seeds.

#### Data analysis

The total number of flowers, mature pods, and seeds per plant were analyzed with generalized linear mixed regression using the log-link function, which assumes a Poisson error distribution. Some plants at the field margins and all plants within the center of fields died, so that data could only be analyzed for marginal plants; this yielded a total sample size of 20 plants. Spraying and the three fields were fixed factors, while a random factor grouped each pair of plants consisting of a sprayed and an unsprayed plant. All analyses were carried out using the statistics software R (R Development Core Team 2012).

## Results

### Field Experiment I

**Figure 2:**
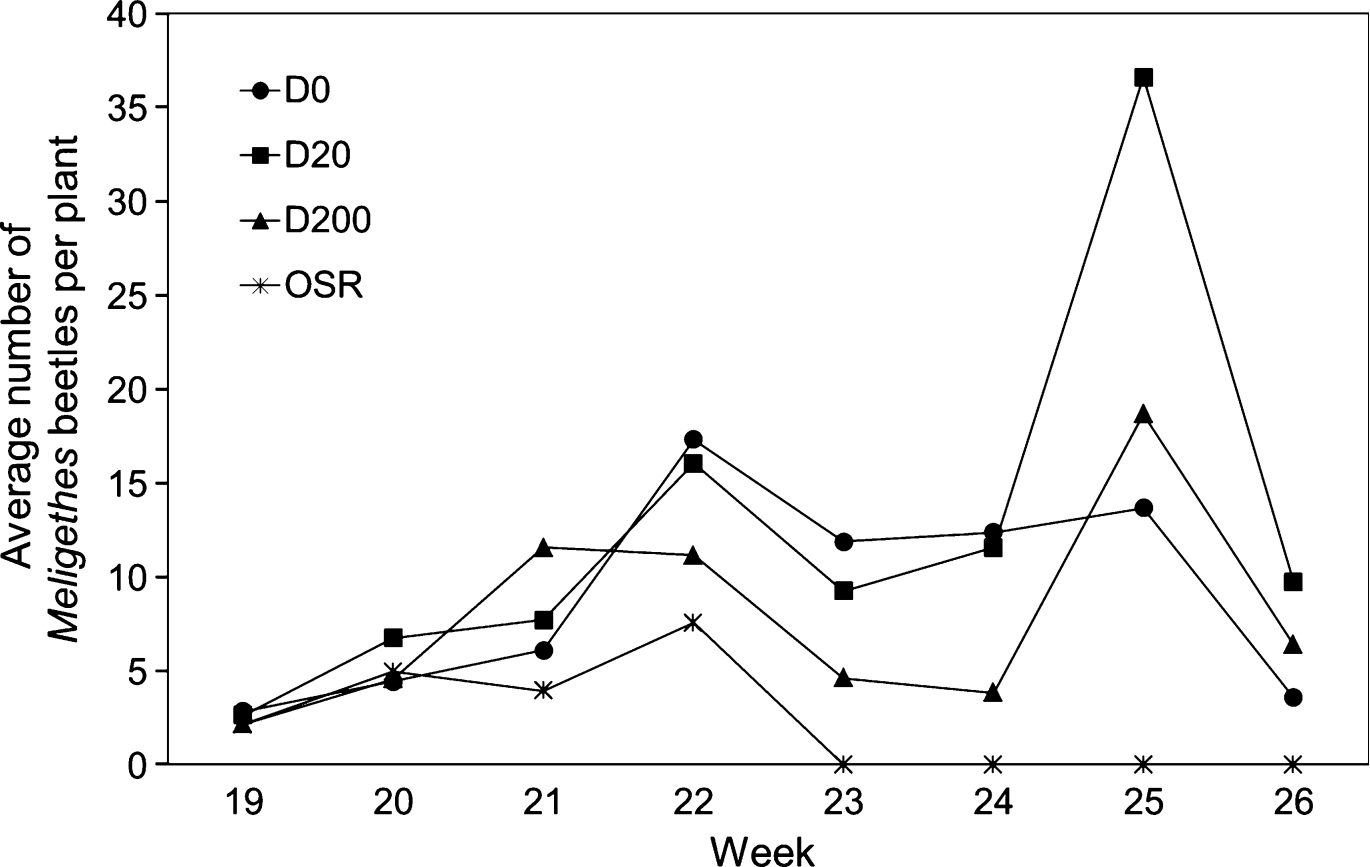
Average number of *Meligethes* beetles on unsprayed *Raphanus raphanistrum* plants at the three different distances (D0 = 0 m, D20 = 20 m, D200 = 200 m) and on oilseed rape (OSR).

The average number of *Meligethes* beetles on oilseed rape plants increased from around two per plant at week 19 to a peak of 8 per plant at week 22. No beetles were found on oilseed rape plants after week 23, when the flowers had withered and oilseed rape pollen was no longer available (Fig. 2). The numbers of *Meligethes* on *R. raphanistrum* plants were generally higher than those on oilseed rape plants, with similar trends at all distances. Numbers increased steadily until week 22, when they reached a first peak that corresponded with the peak on oilseed rape; subsequently, the number of beetles decreased (Fig. 2). These declines were then followed by a strong increase in the plants 20 (D20) and 200 m (D200) away and only a moderate increase in the plants adjacent to the field (D0). By far, the highest density of beetles was observed in week 25 at D20, when there was an average of 37 beetles per plant.

**Figure 3:**
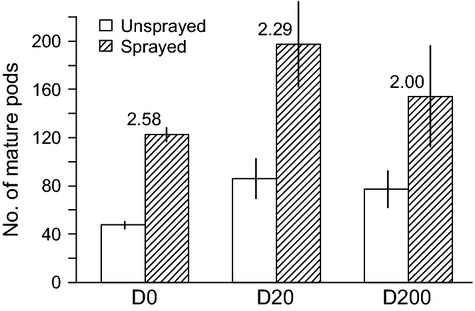
Number of mature pods per *Raphanus raphanistrum* plant as affected by spraying for distances of 0 (D0), 20 (D20), and 200 m (D200) from oilseed rape fields. Displayed are means across all sites ± 1SE. Values above bars are the ratio of the number of sprayed divided by the number of unsprayed mature pods.

*Raphanus raphanistrum* plants that were sprayed with insecticide produced more than twice as many mature pods as unsprayed plants at all distances from the fields (Fig. 3), resulting in a highly significant spraying effect (Table 2). The effect of spraying was highly significant at all distances (*P* < 0.001 each), but varied with distance from the fields (Table 2: distance x spraying interaction), being greatest at D0 and least at D200 (Fig. 3). The spraying effect on the number of mature pods as well as the effect of distance also varied among sites (Table 2: site x spraying and site x distance interactions, see electronic appendix Figures S1 & S2), but the effect of spraying was significant at all sites and there was no significant overall influence of site on the number of mature pods produced (Table 2).

**Table 2:** Effects of site, distance from field, and spraying treatment on the number of pods of *Raphanus raphanistrum* (log-transformed)

Variable	Df_effect_	χ^*2*^	*P* (≥χ^*2*^)
Site	4	2.80	0.592
Distance	2	7.20	0.027
Spraying	1	112.84	< 0.001
Site x distance	8	64.68	< 0.001
Site x spraying	4	10.89	0.028
Distance x spraying	2	14.05	< 0.001

Data were analyzed with linear mixed regression, and inference is based on likelihood ratio tests.

Given that the mean numbers of seeds per mature pod were similar for sprayed plants and unsprayed plants (mean values 5.0 and 4.7, respectively, no significant difference), it can be concluded that seed production was approximately 50% higher in sprayed plants.

### Field Experiment II

**Figure 4:**
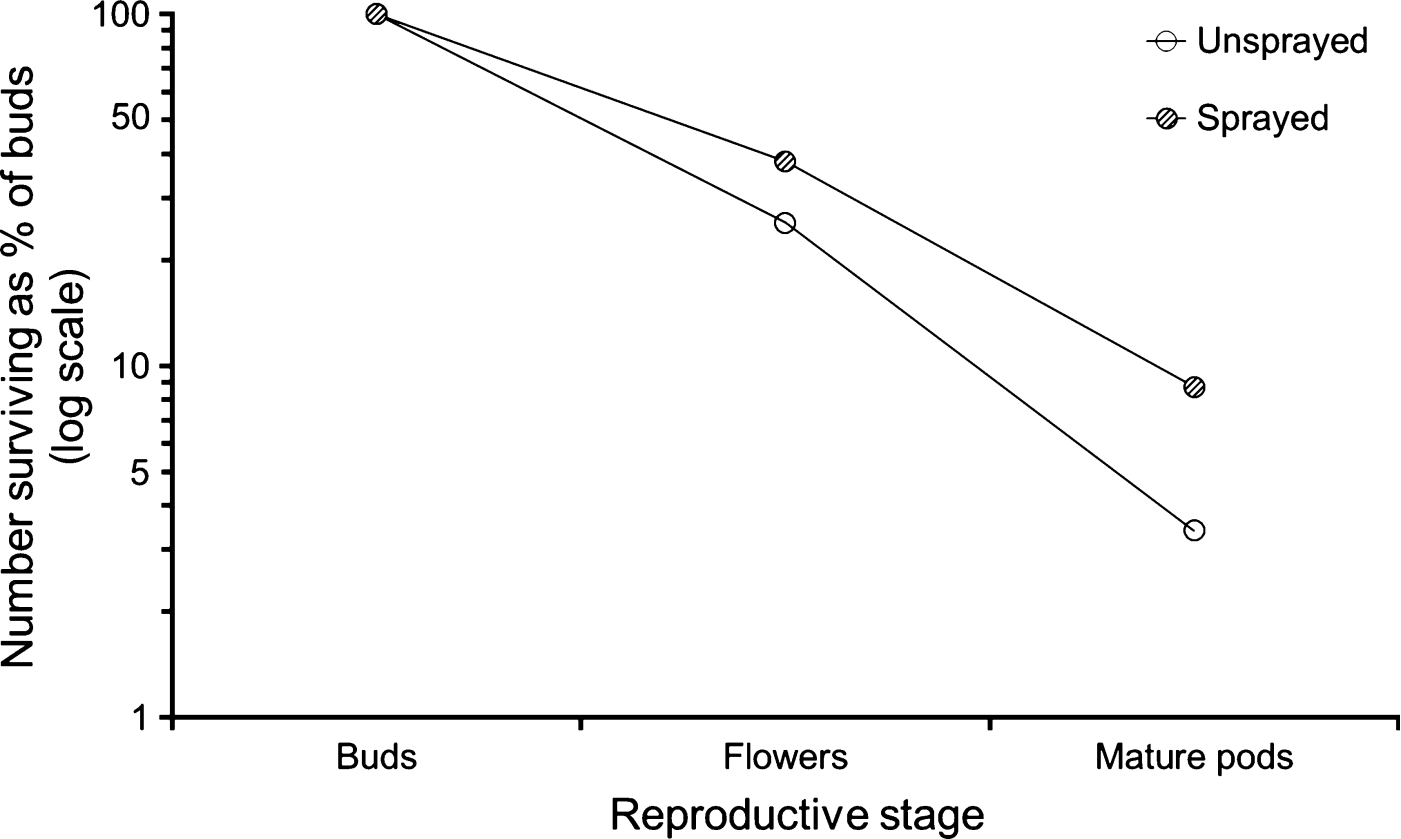
Survivorship curve of reproductive success in *Raphanus raphanistrum* scaled to 100 buds per plant (=100%).

While the total number of flowers was not significantly affected by the spraying treatment, the number of mature pods and seeds clearly was (Table 3). In absolute terms, total number of mature pods was about three times as high in sprayed than in unsprayed plants (74 ± 34.5 vs. 25 ± 5.9; mean across all three fields ± 1 SE), and the total number of seeds per plant was about twice as high (202 ± 104.8 vs. 95 ± 26.5). The respective values for sprayed and unsprayed flowers were 428 ± 174.9 vs. 252 ± 54.2. In Fig. 4, these data are presented in the form of survivorship curves; these show that 38% of buds from sprayed plants became flowers, of which 8.7% developed into mature pods, while the equivalent values for unsprayed plants were 26% and 3.4%, respectively.

**Table 3:** Effect of spraying and field site on reproductive structures of *Raphanus raphanistrum* plants

		Flowers		Mature pods		Seeds	
				
Variable	Df_effect_	*χ*^*2*^	*P* (≥*χ*^*2*^)	*χ*^*2*^	*P* (≥*χ*^*2*^)	*χ*^*2*^	*P* (≥*χ*^*2*^)
Field	2	2.32	0.314	3.59	0.166	4.34	0.114
Spraying	1	1.33	0.250	8.91	0.003	4.64	0.031
Field x spraying	2	1.48	0.477	0.18	0.913	0.22	0.894

Data were analyzed with generalized linear mixed regression, and inference is based on likelihood ratio tests.

## Discussion

The density of the specialist herbivore *Meligethes* spp. was generally higher in *R. raphanistrum* than in oilseed rape plants, but their numbers were strongly influenced by the location and phenology of the crop. The highest densities were recorded in *R. raphanistrum* plants within 20 m of oilseed rape fields, although many insects were also found in plants at 200 m, indicating that beetles are highly mobile. Furthermore, infestation was high not only during the flowering period of oilseed rape, but for several weeks afterward, with the highest levels of infestation occurring in week 25, after the crop had withered. Larvae of the *Meligethes* beetles are known to pupate in the soil about 1 month after egg-laying and to emerge about 2 weeks later (Hokkanen 2000). As *Meligethes* spp. started to move into oilseed rape fields during week 19 (Fig. 2), the peak in week 25 probably represented a new generation of beetles that had emerged and left the crop in search of food.

Both experiments clearly showed that protecting *R. raphanistrum* plants against *Meligethes* spp. significantly increased their fitness. Thus, in the first experiment, pod production and seed production of sprayed plants was at least 50% higher than that of unprotected plants, with the effect being largest for plants close to an oilseed rape field. In comparison, the observed increase in seed production is similar to the 55% increase found in Bt sunflowers (Snow et al. 2003). The second experiment showed that protection from *Meligethes* spp. favored the development of the reproductive stages from buds to pods, and especially the transition from buds to flowers.

According to apparent competition theory, one prey species can be suppressed or even excluded from an area if another prey species supports a very high density of enemies common to both species (Holt and Lawton 1994). In our study system, it seems probable that the very large populations of *Meligethes* spp. supported by the oilseed rape crop have a negative effect on populations of *R. raphanistrum,* especially in areas where oilseed rape is cultivated on a large scale. As shown in our study, *Meligethes* beetles substantially reduce the reproductive output of *R. raphanistrum*, possibly limiting its population. However, despite the expansion of the cultivated oilseed rape area in recent decades in Swiss agricultural systems, *R. raphanistrum* persists in these areas, although it is never very abundant, in the sense that it becomes a serious weed. We cannot say, however, whether this already low abundance is further decreasing due to the increased cultivation of oilseed rape, or whether it has reached an equilibrium level. In support of the latter possibility, models show that spatial heterogeneity and behavioral responses by the predator (here, a herbivore) can provide refuges for a prey (here, a host plant), thereby enabling coexistence (Bonsall 2003). Indeed, Thies et al. (2003) found that the rate of herbivory by *M. aeneus* on its host plants depends on the proportion of oilseed rape in a landscape. Alternatively, *R. raphanistrum* may be able to persist because it has a long flowering period from spring until fall, whereas winter oilseed rape plants only flower for a few weeks during April and May. Thus, it may be able to produce enough seeds later in the season, when *Meligethes* beetles have moved to their hibernation sites, to maintain its population.

In view of these results, it is relevant to ask what would happen if *R. raphanistrum* were to acquire resistance to *Meligethes* spp. by crossing with transgenic oilseed rape. One possible scenario is provided by the present situation in southern Australia, where *R. raphanistrum* was introduced accidentally in the middle of the 19th century. Unlike in Europe, *Meligethes* beetles are absent in Australia and there appears to be no equivalent natural control (Austin et al. 2004), with the consequence that *R. raphanistrum* has become a serious weed, especially in oilseed rape fields (Murrumbidgee Catchment Management Authority 2008). In our study area, however, where *Meligethes* beetles are common, there would be strong selection for increased resistance against *Meligethes* beetles (Marvier and Kareiva 1999). Indeed, transgenic resistance (e.g., Bt) might prove more effective than insecticides in controlling *Meligethes* beetles because transgenic plants produce insecticidal toxins from the seedling stage until plant senescence (e.g., Koziel et al. 1993), whereas insecticides provide only temporary resistance. Thus, there is good reason to suppose that genotypes of *R. raphanistrum* containing insect (in particular coleopteran) resistance transgenes from oilseed rape could spread rapidly. This is supported by theoretical models showing that the rate of spread of a novel allele in a population is governed by the selective advantage that it confers, and not by the rate of hybridization (Rieseberg and Burke 2001). Although our simulation experiments do not allow for any conclusion on the fitness cost in *Raphanus* hybrids due to transgene expression, experiments with transgenic insect-resistant rice hybrids showed negligible costs under high insect pressure and only a small cost under low insect pressure (Yang et al. 2011).

Whether an insect-resistant transgene from oilseed rape would spread rapidly within the population of *R. raphanistrum* and in turn the population would increase after adaption of transgenic insect resistant oilseed rape cannot be answered conclusively from the observed increase in seed production, as many other factors might prove important. For example, if beetle populations were substantially reduced by transgenic oilseed rape, then the reduced herbivore pressure could lead to an increase in population of *R. raphanistrum* without gene transfer. Of course, such an increase would also depend on a possible population increase of other competitors. If herbivore pressure remained high on *R. raphanistrum* despite transgenic oilseed rape (e.g., due to low adoption of transgenic varieties by farmers), hybrids between transgenic insect-resistant oilseed rape and *R. raphanistrum* might have a selective advantage (assuming, of course, that the Bt trait was sufficiently expressed in the hybrid plants and fitness costs due transgene expression were negligible). Under these circumstances, the transgene might spread rapidly in the *R. raphanistrum* population, despite the fact that outcrossing between oilseed rape and *R. raphanistrum* is rather rare (Darmency et al. 1998).

In the light of anthropogenic impacts increasingly driving ecological and evolutionary processes, especially in agro-ecosystems, Thrall et al. (2011) propose that predictive frameworks based on evolutionary models should be used as pre-emptive management tools. The relatively simple experiments presented in this article provide necessary information to build such a predictive framework by characterizing the relevant biotic interactions involved in the studied system. In this way, the potential consequences of transgene flow of an insect-resistance trait can be evaluated, and the results used to make informed management decisions. For environmental risk assessment of transgene flow, in particular, we conclude that a shift in emphasis is needed from determining the rate and success of gene transfer to understanding the selection pressures in particular environments. Any crop is part of a larger community of organisms including wild plants and the many insect species that move between cultivated and wild plants. It is only by investigating the interactions among these organisms that we can detect some of the indirect effects of the escape of transgenes.
